# Navigating conflict and difference in medical education: insights from moral psychology

**DOI:** 10.1186/s12909-018-1383-z

**Published:** 2018-11-22

**Authors:** Samuel Paros, Jon Tilburt

**Affiliations:** 1 College of Medicine, Medical University of South Carolina, 171 Ashley Ave, Charleston, SC 29425 USA; 2 Biomedical Ethics Research Program, Division of Health Care Policy and Research, Division of General Internal Medicine, Mayo Clinic, 200 1st Street SW, Rochester, MN 55905 USA

## Abstract

Medical students and educators face a myriad of complex moral disagreements and conflicts both in preclinical and clinical training environments. Inability to deal with these conflicts effectively and compassionately can lead to undesirable consequences and threaten important relationships in high-stakes healthcare environments. We suggest that the integration of moral psychology into medical education can help trainees and faculty constructively respond to behavior they may find immoral or misguided. Here we focus on the application of Moral Foundations Theory (MFT), which demonstrates how the instantaneous gut reactions which guide reactionary behavior can be categorized into six foundational categories. These categories offer psychological explanations for human behavior which can help medical trainees and professionals navigate challenging moral conflicts.

## Background

Political, social, and cultural divisions are as visible now as ever both in society and in healthcare [1]. Healthcare environments present high-stakes circumstances in which cooperation among patients and care teams is crucial. Care providers strive to ensure that conversations are compassionate and respectful regardless of a clinician or patient’s background or identity. Yet providing quality health care for a person who in a clinician’s opinion holds objectionable perspectives can be difficult. Additionally, patients may not accept medical advice from a clinician whose lifestyle or culture they do not respect. Indeed, political differences between physicians have the potential to determine the type of advice they give to patients [2]. Thus ideological and cultural differences have the potential to threaten high quality care delivery. To the extent that these situations arise during medical training, medical students are often caught in the middle [3, 4]. Medical students face a wide range of differing moral opinions in their training and must navigate and learn to respond constructively to those differences.

We propose that creating a space for moral psychology in medical training will improve physicians’ in-training abilities to connect with and care for diverse patient populations with the competency called for in Liaison Committee on Medical Education (LCME) curricular standards [5]. Identifying one’s own moral intuitions and exploring how they may be similar to or different from those of others could prove crucial while delivering quality care in our culturally fragmented society. Here we briefly describe and then apply one particular theory of moral psychology that if integrated into medical education will ultimately cultivate self-awareness and compassion necessary for delivering high quality 21st century care.

## Moral psychology: Social intuitionism

Social Intuitionism and Moral Foundations Theory (MFT) are schools of thought in the field of moral psychology which aim to explain the evolutionary and cultural basis of differences in moral intuition that we find particularly helpful for understanding moral conflict in medical education [6]. Earlier theories of morality by Kohlberg and others favored the role of formal reasoning [7]. In contrast Moral Foundations Theory, through its chief proponent, Jonathan Haidt, has postulated that humans react to others with instantaneous gut reactions, so called moral intuitions. These innate intuitions co-evolved with cultural and social practices to form a group of reactions that can be categorized into care/harm, fairness/cheating, loyalty/betrayal, authority/subversion, sanctity/degradation and liberty/oppression (Table 1). On this view, people provide after-the-fact (so called “post-hoc”) justifications for their emotionally-motivated reactions, which gives the illusion (to themselves and others) of being well-reasoned [8, 9]. Haidt uses the metaphor of ‘the elephant and the rider’ to depict the relationship between moral emotions and reason. In the moral life, a heavy visceral subcortical system of reactions lumbers along with an unspoken yet powerful strength and momentum (elephant) below the conscious attention (rider). The elephant’s rider, reason, exists to justify and after the fact adjust the direction already set by the beast below (as it were) with arguments and principles [9]. For example, in situations of thoughtful contemplation, the rider calmly works with the elephant and steers him in a desired direction using reason and arguments. When emotions run high, the elephant may forget about the rider, instead deciding to run off in a direction he feels is important in that moment. Haidt’s account of moral psychology borrows from assumptions in the moral philosophy of thinkers like David Hume, who asserted that reason’s influence over our visceral reactions is limited [10].

**Table 1:** An adapted version of the origins, triggers and characteristics of the six moral foundations from The Righteous Mind by Jonathan Haidt [9]

	Care/ harm	Fairness/ cheating	Loyalty/ betrayal	Authority/ subversion	Sanctity/degradation	Liberty/oppression
Adaptive challenge	Protect and care for children	Reap benefits of two-way partnerships	Form cohesive coalitions	Forge beneficial relationships within hierarchies	Avoid contaminants	Ensure freedom from dominant members of society
Original triggers	Suffering, distress, or neediness expressed by one’s child	Cheating, cooperation, deception	Threat or challenge to group	Signs of dominance and submission	Waste products, diseased people	Bullies, tyrants, signs of attempted domination
Current triggers	Baby seals, cute cartoon characters	Marital fidelity, broken vending machines	Sports teams, nations	Bosses, respected professionals	Taboo ideas (communism, racism)	Government, taxation, big corporations
Characteristic emotions	Compassion	Anger, gratitude, guilt	Group pride, rage at traitors	Respect, fear	Disgust	Righteous anger/reactance
Relevant virtues	Caring, kindness	Fairness, justice, trustworthiness	Loyalty, patriotism, self-sacrifice	Obedience, deference	Temperance, chastity, piety, cleanliness	Liberty, freedom, justice

The categories that comprise these foundational gut reactions in moral foundations theory are divided up into care/harm, fairness/cheating, loyalty/betrayal, authority/subversion, sanctity/degradation, and liberty/oppression. These evolved categories characterize the “right/wrong” visceral compass of the elephant evolved to promote safety and social cohesion. In modern society, groups differ in the amount of value they assign to each of these ancient moral foundations. For example, political liberals typically prioritize care/harm and fairness/reciprocity relative to other categories, while political conservatives preserve all six foundations in their reactions [11]. This may be thought of as two different elephant breeds with different visceral navigation systems. Within healthcare, differences in physicians’ views on abortion and physician aid-in-dying seem to track with the weight they place on purity/sanctity in particular [12, 13]. We think there is a pedagogical case for including this kind of content in medical education that may help address the divisive and polarizing climate in which medical students are currently training.

## Case examples

Two recent cases in the authors’ collective experience, one from pre-clinical medical school training, and one from clinical training settings, illustrate challenges medical students and educators commonly face in navigating and responding constructively to moral differences in their training. We then describe how Moral Foundations Theory can be employed to help one navigate and respond to such differences.

### Preclinical case

*As part of their first year curriculum, a class of medical students is required to attend a sexual health lecture series by a visiting professor. The presentation includes graphic imagery of (what some in mainstream society would describe as) explicit, deviant and taboo sexual behaviors. The lecture series aims to inform students about a wide range of sexual behaviors lest they be unaware or inadvertently unprepared or uncomfortable when encountering patients who disclose such behaviors. One student with strong religious beliefs refuses to attend the lecture, citing her moral opposition to even viewing such content, and seeks to achieve the required competencies of the unit through alternative educational means. This prompts conflict with the course director, and the case escalates to the dean’s office.*

### Clinical case

*A medical student on his hematology/oncology rotation is helping establish a care plan for an older male patient recently diagnosed with pancreatic adenocarcinoma. When the student returns to the patient’s room to check in with the family, the patient’s wife interjects: “I have one request. I just don’t want him to be treated by one of those foreign doctors.” The rest of the family members nod their heads in approval.*

These routine cases demonstrate how deeply held morals bump up against established institutional norms. Although we may have our own opinions about the rightness and wrongness of the behaviors described in these cases, here we will refrain from describing our own positions and instead invite our readers to suspend their own immediate judgments long enough to consider how moral psychology can inform a constructive approach to addressing moral differences in medical education. Below we discuss these cases and briefly describe how and why we think moral psychology particularly insights from Moral Foundations Theory might augment medical school learning to help students constructively face and ultimately address moral differences, particularly when one finds some behavior/beliefs “offensive.”

If the visceral account of moral difference described by social intuitionism is accurate, medical trainees are not immune to this wiring. Furthermore, given current practices in recruiting diverse student classes, we should expect differences in moral intuitions among medical student cohorts and in the complex organizations in which they train. Arguably, moral diversity is crucial to fostering a diverse educational environment, yet cultural diversity often brings with it heterogeneity in how the various dimensions of the moral life are weighed. This poses organizational and curricular challenges. Organizationally, healthcare institutions including academic medical centers that espouse a “diversity and inclusion” mandate, cannot unreflectively and implicitly enforce a worldview that privileges a particular constellation of moral intuitions while unwittingly disparaging others. To do so would undermine the very cause they espouse (and yet some minimal standards of civility must nevertheless be maintained and cultivated).

In our preclinical case, the student’s refusal to view what may be described as sexually deviant behavior is likely based on moral intuitions that prioritize purity and sanctity. According to moral foundations theory, the foundation of purity/sanctity views the human body as a temple worth preserving and protecting from desecration and disease. Historically, this foundation has likely fostered social cohesion and survival advantages over the course of human existence [9, 14]. To ignore this deep wiring in learners is dangerous and short sighted. In this case, faculty and deans should at least ask the question, “must all learners regardless of their antecedent cultural and religious wiring be exposed to differences in the same way in order to achieve the desired competencies?” Does not a commitment to navigating diversity and moral difference compel us to attempt a charitable interpretation to this learner’s deep aversion to the objectionable course content?

In the clinical case, let us suppose that the student strongly disagrees with the patient’s family’s perspective on foreign-trained doctors, finding their expressions offensive and bigoted. For a student who views the spouse and family behavior offensive/inappropriate, it does not follow that refutation or confrontation are the only effective, high-integrity responses. Responding too directly could prove counterproductive to the care of the patient. Moral foundations theory offers potential for a more charitable explanation of the views of both the student and the patient and family.

The foundation of fairness/equality is likely fueling the student’s refusal to consider that foreign-trained doctors may be less competent. This foundation emphasizes the importance of promoting a fair and just society for all groups of people, and privileging this foundation is understandable. The family’s wariness of accepting care from a foreign-trained doctor may be a result of favoring the foundation of in-group/loyalty, a mechanism evolved to protect certain groups (i.e. “Americans”) from potentially dangerous outsiders (i.e. “foreigners”). In addition, the foundation of harm/care seems to be prioritized for the patient and their family when faced with a dire prognosis. As such, it is reasonable to assume that being forced into such a vulnerable state full of many unsafe feelings and reactions will lead the patient and family to “hunker down,” reacting in ways that get expressed in terms of what the student considers bigoted remarks about preference in doctor. As a matter of process, both have visceral reactions that deserve a charitable description prior to formulating a collegial response that might involve clarification or refutation. A response plan devised with the assistance of an attending faculty member, that accounts for an appreciation of the visceral nature of many moral judgements can inspire the student to address the concerning behavior of the patient and family, but do so with a degree of charitability toward the family’s viewpoints. Ideally, this would aid the student in developing a more constructive working relationship with the family and spouse. However, it should be noted that charitability does not equate to agreement, and appreciating the psychological roots of another’s ethical judgments does not demand that we change our own.

## The pedagogical case for moral psychology in medical education

Students must learn to work with patients and colleagues across the political and cultural spectrum, exposing them to ideas, beliefs, and lifestyles that are in direct conflict with their own. Moral psychology has the potential to shape how best to introduce students to differences that first cultivate awareness of the moral matrices that govern their own behavior, allowing them to build the skills necessary to identify their own emotions that drive their judgments of right and wrong. Then a student could, and arguably should, be provided with the psychological tools to appreciate what others view as normal or deviant, moral or immoral.

We view the kind of antecedent self-awareness, compassion and ultimately collegiality we are calling for as a form of moral maturity for practicing medicine in the diverse realities of the 21st century. Many current medical ethics curricula primarily focus on the application of ethical principles to case studies while providing time for thoughtful discussion and reflection [15]. Some moral foundations, too, are strikingly in line with the principles of bioethics. Care/harm is more or less represented by beneficence and non-maleficence, while fairness/cheating is similar to justice [12]. Other foundations like loyalty/betrayal, authority/subversion, and sanctity/degradation do not directly overlap with ethical principles. Behavior motivated by such foundations (as illustrated by our case examples) thus has the potential to confuse students and clinicians whose understanding of ethics is based on a traditional curriculum alone. From a curricular perspective, asking students to dispassionately abide by a specific set of purportedly universal abstract ethical principles of the intellectual rider without first appreciating the distinct underlying moral elephant beneath a particular culture or faith is short-sighted.

Education on moral foundations theory can be thought of as similar to the teaching of anatomy and physiology in the preclinical years of medical school. Much like learning anatomy gives students a foundation in human body composition and function to aid them in their understanding of disease, moral psychology provides a robust framework of philosophical and psychological reasoning which can inform medical students as they face manifestations of moral difference in practice.

Identifying the empirical reality of multiple foundations in moral intuitions does not mean that all categories are equally valid or necessarily must be respected. There could be a moral fact of the matter to which some groups and traditions have more closely approximated. Rather, our point is that if we seek to achieve greater insight and capacity for medical students to navigate medicine in a pluralistic society, that navigation should be undergirded with an awareness of how deeply and differently people may be wire on matters of moral difference.

Achieving the type of moral maturity we suggest can be difficult when engaging with views that present an affront to a clinician’s deeply engrained, foundational beliefs whether they be a commitment to human rights, beliefs about the impurity of certain practices or tolerance of diverse ethnic and religious groups. Strongly held beliefs can sometimes lead individuals to become convinced of a ‘myth of pure evil’ which identifies those who are opposed to or simply do not align themselves with said beliefs as having poor moral character. We argue that introducing concepts focused on the moral psychology describing underlying moral differences could instead help students and practitioners better appreciate the humanity of those with whom they are in contention with, helping foster compassion in the process.

The integration of MFT material into the medical school curriculum would be championed by faculty members invested in teaching students about the complexities of the doctor-patient relationship. It may prove difficult to enlist the help of faculty in promoting the addition of social science content into medical schools, which is why emphasizing the clinical and case-based applications of such material is essential in generating interest. Educators in mental health specialties which utilize psychological theory such as psychiatry and neurology may serve an important role in the introduction of ideas such as moral foundations theory. Moreover, as the practical relevance of this material takes hold clinically, arguably educators as well as administrators may benefit from these same insights.

Critics might rightly argue there is no current evidence that including moral psychology in medical education improves any desirable educational outcome. Admittedly we should not expect moral disagreements to be suddenly solved by these curricular changes. However, we would argue that our suggestions form a plausible and testable pedagogical hypothesis on improving medical training which can and ought to be investigated including if necessary, development of difference outcome measures. This hypothesis would creatively blend the objectives of Biomedical, Behavioral and Social Sciences (7.1), Medical Ethics (7.7) and Cultural Competence (7.6) in the current LCME standards and do so in a way that takes seriously the politically-charged social divisiveness of our moment [5]. Ultimately, by embracing awareness of how the moral worlds of humans are created by and stimulated by experiences, medical students are more likely to identify practical, compassionate solutions while allowing them to maintain their sense of integrity as moral agents. In order to develop a more robust understanding of the practical effects of moral psychology education, empirical studies testing self-awareness and compassion in medical student cohorts through moral foundations theory could be tested.

## Conclusion

Resurgent social, cultural and political differences in healthcare require adjustments in medical education pedagogy. Moral psychology can foster curiosity and flexibility about deep, intuitive moral differences that may arise in medical education to constructively address conflict without fueling divisiveness. Introducing moral psychology into medical education as a practical tool for greater self-insight and greater compassion for patients, families, and colleagues with whom we differ will provide future medical practitioners with the cultural and interpersonal self-awareness to listen to, learn from, and respond to a diverse array of moral viewpoints while upholding the high ideals of medicine in the hard work of 21st century healthcare.
